# Economic evidence for the prevention and treatment of atopic eczema: a protocol for a systematic review

**DOI:** 10.1186/s13643-016-0262-0

**Published:** 2016-05-27

**Authors:** Tracey Helen Sach, Emma McManus, Christopher Mcmonagle, Nick Levell

**Affiliations:** Health Economics Group, Norwich Medical School, University of East Anglia, Norwich, NR4 7TJ UK; Dermatology Department, Norfolk and Norwich University Hospitals NHS Foundation Trust, Colney Lane, Norwich, NR4 7UY UK

**Keywords:** Eczema, Economics, Costs, Health-related quality of life, Cost effectiveness

## Abstract

**Background:**

Eczema, synonymous with atopic eczema or atopic dermatitis, is a chronic skin disease that has a similar impact on health-related quality of life as other chronic diseases. The proposed research aims to provide a comprehensive systematic assessment of the economic evidence base available to inform economic modelling and decision making on interventions to prevent and treat eczema at any stage of the life course. Whilst the Global Resource of Eczema Trials (GREAT) database collects together the effectiveness evidence for eczema, there is currently no such systematic resource on the economics of eczema. It is important to gain an overview of the current state of the art of economic methods in the field of eczema in order to strengthen the economic evidence base further.

**Methods/design:**

The proposed study is a systematic review of the economic evidence surrounding interventions for the prevention and treatment of eczema. Relevant search terms will be used to search MEDLINE, EMBASE, Database of Abstracts of Reviews of Effects, Cochrane Database of Systematic Reviews, Cochrane Central Register of Controlled Trials, National Health Service (NHS) Economic Evaluation Database, Health Technology Assessment, Cumulative Index to Nursing and Allied Health Literature, EconLit, Scopus, Cost-Effectiveness Analysis Registry and Web of Science in order to identify relevant evidence. To be eligible for inclusion studies will be primary empirical studies evaluating the cost, utility or full economic evaluation of interventions for preventing or treating eczema. Two reviewers will independently assess studies for eligibility and perform data abstraction. Evidence tables will be produced presenting details of study characteristics, costing methods, outcome methods and quality assessment. The methodological quality of studies will be assessed using accepted checklists.

**Discussion:**

The systematic review is being undertaken to identify the type of economic evidence available, summarise the results of the available economic evidence and critically appraise the quality of economic evidence currently available to inform future economic modelling and resource allocation decisions about interventions to prevent or treat eczema. We aim to use the review to offer guidance about how to gather economic evidence in studies of eczema and/or what further research is necessary in order to inform this.

**Systematic review registration:**

PROSPERO CRD42015024633

**Electronic supplementary material:**

The online version of this article (doi:10.1186/s13643-016-0262-0) contains supplementary material, which is available to authorized users.

## Background

In the UK, the lifetime prevalence of eczema is estimated to be between 16 and 20 % making it the commonest inflammatory skin condition in children, and it has been increasing in “western style” environments [1–3]. In the UK, the age-sex standardised incidence and lifetime prevalence of eczema has increased between 2001 and 2005 from 9.58 per 1000 to 13.58 per 1000 patients and 77.78 per 1000 to 115.26 per 1000, respectively [4]. Up to 50 % of childhood cases will experience recurrence in adulthood [2]. Eczema is largely managed in primary care. Skin conditions are the commonest new reason patients consult their GP [5]. Eczema has been found to have a similar impact on health-related quality of life as other common childhood conditions such as asthma and diabetes [6]. Eczema impacts quality of life by causing itching, sleep loss and social stigma for the child. Families may also suffer from sleep loss and time taken off work to accompany children to health appointments [7]. The condition is associated with atopy so children with the condition are more likely to develop asthma and allergic rhinitis [8]. It is also believed that eczema has large cost implications. For instance, in 1995–1996, the total annual UK cost of eczema in children aged 5 years or younger was estimated as £47 million (£80 per child) [9]. Looking at a broader age range, the UK total annual cost was estimated to be around £465 million, of which £125 million were National Health Service (NHS) costs, £297 million costs incurred by patients and £42 million by society in terms of lost productivity (price year not reported) [10]. These UK-based estimates of the total annual UK costs of eczema are now dated, the estimates were based on small samples [146 in [10] and 1523 in [9]], and the range of treatments available has increased and is likely to increase in the future with the addition of new biologics [11].

Despite eczema being common, there remain many unknowns about how to prevent and treat the condition. The James Lind Alliance (JLA) [12] Priority Setting Partnership (PSP) on eczema illustrates this (http://www.jla.nihr.ac.uk/priority-setting-partnerships/eczema and [13]). The JLA facilitates disease specific PSPs that bring together patients, carers and health professionals to identify and prioritise research for the treatment uncertainties of the disease of interest (http://www.jla.nihr.ac.uk/). The eczema PSP was established in 2010 with partners drawn from academic, NHS and charitable sectors and resulted in 14 treatment uncertainties being prioritised [13].

In order to draw together the effectiveness evidence of interventions for eczema, the Global Resource of Eczema Trials (GREAT) [14] database was established [15] and includes details of over 600 systematic reviews and randomised controlled trials. It does not, however, identify or bring together the economic literature on eczema, and thus, this review attempts to do this. There is likely to be less economic evidence, compared to effectiveness data, for eczema. Indeed, the English National Institute of Health and Care Excellence (NICE) has only considered economic models for two areas of eczema care: an educational intervention for those with eczema aged under 12 [16] and tacrolimus and pimecrolimus for atopic eczema [17]. It is, therefore, important to identify the current state of economic evidence addressing eczema in order to inform the design of future economic research in the area.

The proposed systematic review will address the following four research questions:

1. What type of health economic evidence has been used in the evaluation of the prevention and treatment of atopic eczema?
2. Are interventions to prevent and treat atopic eczema cost effective?
3. What is the quality of the health economic evidence for the prevention and treatment of atopic eczema?
4. What are the current gaps in the existing research?

## Methods/design

### Protocol and registration

The Preferred Reporting Items for Systematic Reviews and Meta-Analyses Protocols (PRISMA-P) statement recommendations were used to develop the methods for this systematic review (see Additional file 1) and will be used in reporting the results from the study [18]. This protocol has been registered in the International Prospective Register of Systematic reviews (PROSPERO) CRD42015024633. Should protocol amendments be necessary, these will be documented including details of the date, changes made and the rationale for changes.

### Literature search

The following electronic databases will be searched: MEDLINE, EMBASE, Cumulative Index to Nursing and Allied Health Literature, Cochrane Central Register of Controlled Trials, Database of Abstracts of Reviews of Effects, Cochrane Database of Systematic Reviews, NHS Economic Evaluation Database, EconLit, Scopus, Health Technology Assessment, Cost-Effectiveness Analysis Registry and Web of Science. There will be no restriction imposed on the search from date; therefore, the literature search will go from the earliest records within each database, to the single day that the search was conducted on. Search results will be downloaded to Endnote version X7 where duplicates will be identified and removed.

Reference lists of potential eligible studies, reviews, guidelines or other sources will be screened for additional literature. Authors of published abstracts and conference proceedings will be contacted by email to establish if a full paper has since been published in the grey literature.

The search strategy was guided by The Cochrane Collaboration Handbook [19] and the Centre for Reviews and Dissemination guidelines for systematic reviews [20]. Specifically, search terms used in other atopic eczema systematic reviews were used to inform the clinical search terms [21]) and were also informed by the clinical author on the paper: NL. The economic terms were similarly devised, by consulting relevant guidelines [22, 23]. The complete search strategy (with interface and coverage dates) is available in the Appendix to this protocol.

### Eligible studies

A study will be included if it reports primary data on cost and/or outcome (utility or willingness to pay) data on atopic eczema. The primary interest is in full economic evaluations (cost effectiveness, cost utility, cost benefit and cost minimisation) although other partial economic evidence will also be included where the study has an explicit economic objective, this is likely to include cost-consequence analyses, cost analyses, utility assessment or willingness to pay/accept studies. There will be no restriction on the study designs used in the economic studies so, for example, economic studies conducted alongside randomised controlled trials, as part of observational studies, or as decision model-based analyses will be included. Nor will there be any restrictions on type of setting. The search was undertaken on the 9th October 2015, so only studies published before this date are included. Only full text articles published in the English language will be included, abstracts and letters will be excluded. Where two or more studies appear to be reporting on the same dataset or using the same model, the most comprehensive paper will be included unless each paper reports on a different aspect or in relation to a different jurisdiction/population (in the case of modelling studies).

## Data collection

### Study selection

Two independent reviewers will assess the titles and abstracts retrieved in the literature search against our inclusion criteria. In a second stage, full-text articles for those seeming to fit the criteria or where there is uncertainty about relevance will be retrieved and their eligibility assessed according to criteria set out in Table 1. Where disagreements occur a third reviewer will be involved.

**Table 1:** Inclusion and exclusion criteria

	Inclusion criteria	Exclusion criteria
Population	Eczema (synonyms: atopic eczema, atopic dermatitis) ICD-10 code: L20	Populations with other skin diseases other than eczema
		Dyshidrotic eczema, seborrheic eczema, chronic actinic dermatitis, asteatotic eczema, allergic contact eczema, irritant contact eczema, varicose eczema, stasis eczema ICD-10 codes: L21 to L30
Study designs	Studies presenting primary data in the form of a Cost of illness, cost effectiveness, cost utility, cost benefit, cost consequences, cost analysis, utility analysis, contingent valuation	Studies without an explicit economic objective
		Review articles, although the reference list for these will be checked for primary studies that should be included
Outcomes	Utility, QALYs, willingness to pay/accept	
Publication type	Articles available in full text in English	Abstracts, letters, reviews

### Data abstraction and management

Data will be extracted independently by two reviewers and entered into an electronic data extraction form developed in Microsoft Excel, with the third reviewer consulted in case of disagreements that cannot be resolved between the two reviewers. A full list of the extraction fields can be found in Table 2. The data extraction form was piloted, modified (where necessary) and reviewers’ responses calibrated on the basis of two pre-identified studies (one modelling study and one non-model-based paper). The data extracted will be analysed in a narrative/descriptive manner focusing on the methods, results and quality of studies included with the aim of identifying gaps in the evidence, areas of strength and areas in need of methodological improvement.

**Table 2:** Data abstraction fields

General information	
Review ID	
Author, year	
Title	
Reviewer	
Date of review	
Publication type	
Population and setting	
Type of study	
Stated type of economic analysis	
Actual type of economic analysis (if different)	
Country of study	
Study setting	
Population	
Study size	
Method of recruitment	
Recruitment time period	
Inclusion criteria	
Exclusion criteria	
Study design	
Primary intervention	
Secondary intervention(s)	
Comparators	
Time horizon (for follow-up)	
Outcomes	
Outcomes measure (1)	
Method of measurement (1)	
Outcome measure (2)	
Method of measurement (2)	
Outcome measure (3)	
Method of measurement (3)	
Secondary outcome measure(s)	
Method of measurement(s)	
For utility studies: what value set or direct method of measurement has been used?	
Timing of measurements	
Discount rate, outcomes	
Method of dealing with missing data—outcomes	
Resource and cost information	
Cost perspective	
Intervention costs	
Direct cost items	
Method of capturing direct cost items	
Direct cost data sources	
Indirect cost items	
Method of capturing indirect cost items	
Indirect cost data sources	
Resource items collected	
Resource use, recall period	
Method of dealing with missing data—cost	
Price year	
Currency	
Inflation rate, cost	
Discount rate, cost	
Results	
Resource use and costs	
Outcomes	
Reported cost effectiveness	
Appropriateness of ICER	
Sensitivity analysis	
Major result(s)	
Conclusions	
Funding source	
Model specific information	
Type of decision analytic model	
Model perspective	
Model population	
Cohort or individual?	
Model assumptions	
Model exclusions	
Method for dividing disease severity	
Distinction between body/face eczema?	
Interventions included	
Time horizon	
Cycle length	
Value of any parameters used	
Source of parameters	
Software used for model	
Type of sensitivity analysis performed	
Method of model validation	
Author specified limitations	

If the necessary data are available, the results will be discussed as subsets for different age groups (e.g. child/adult/elderly) and/or different skin disease severities and/or world regions and/or health-care settings. Furthermore, as it is expected that included studies will report results in a range of currencies, where possible, results will be reported in the original currency and price year, as well as being converted to UK pounds using the purchasing power parities provided by the Organisation for Economic Co-operation and Development (OECD), inflating to a common price year using the consumer price index, to facilitate the comparison of results across studies.

### Quality assessment and data presentation

Two reviewers will independently evaluate the quality of included studies in order to assess the risk of bias. Studies will be assessed using a published checklist based on a modified version of the Consolidated Health Economic Evaluation Reporting Standards (CHEERS) framework [24] (see Table 3). In addition, model-based economic evaluations will also be assessed using the Phillips criterion [25, 26] (see Table 4 for extraction table). Any discrepancies will be discussed and resolved by a third reviewer.

**Table 3:** CHEERS checklist [24]

	Item no.	Recommendation	Reported on page/paragraph no.	Yes/no/partial/unclear/not applicable
Title and abstract				
Title	1	Identify the study as an economic evaluation or use more specific terms such as “cost effectiveness analysis”, and describe the interventions compared.		
Abstract	2	Provide a structured summary of objectives, perspective, setting, methods (including study design and inputs), results (including base case and uncertainty analyses), and conclusions.		
Introduction				
Background and objectives	3	Provide an explicit statement of the broader context for the study. Present the study question and its relevance for health policy or practice decisions.		
Methods				
Target population and subgroups	4	Describe characteristics of the base case population and subgroups analysed, including why they were chosen.		
Setting and location	5	State relevant aspects of the system(s) in which the decision(s) need(s) to be made.		
Study perspective	6	Describe the perspective of the study and relate this to the costs being evaluated.		
Comparators	7	Describe the interventions or strategies being compared and state why they were chosen		
Time horizon	8	State the time horizon(s) over which costs and consequences are being evaluated and say why appropriate.		
Discount rate	9	Report the choice of discount rate(s) used for costs and outcomes and say why appropriate.		
Choice of health outcomes	10	Describe what outcomes were used as the measure(s) of benefit in the evaluation and their relevance for the type of analysis performed.		
Measurement of effectiveness	11a	Single study-based estimates: Describe fully the design features of the single effectiveness study and why the single study was a sufficient source of clinical effectiveness data.		
	11b	Synthesis-based estimates: Describe fully the methods used for identification of included studies and synthesis of clinical effectiveness data		
Measurement and valuation of preference based outcomes	12	If applicable, describe the population and methods used to elicit preferences for outcomes.		
Estimating resources and costs	13a	Single study-based economic evaluation*:* Describe approaches used to estimate resource use associated with the alternative interventions. Describe primary or secondary research methods for valuing each resource item in terms of its unit cost. Describe any adjustments made to approximate to opportunity costs.		
	13b	Model-based economic evaluation: Describe approaches and data sources used to estimate resource use associated with model health states. Describe primary or secondary research methods for valuing each resource item in terms of its unit cost. Describe any adjustments made to approximate to opportunity costs.		
Currency, price date, and conversion	14	Report the dates of the estimated resource quantities and unit costs. Describe methods for adjusting estimated unit costs to the year of reported costs if necessary. Describe methods for converting costs into a common currency base and the exchange rate.		
Choice of model	15	Describe and give reasons for the specific type of decision analytical model used. Providing a figure to show model structure is strongly recommended.		
Assumptions	16	Describe all structural or other assumptions underpinning the decision-analytical model.		
Analytical methods	17	Describe all analytical methods supporting the evaluation. This could include methods for dealing with skewed, missing, or censored data; extrapolation methods; methods for pooling data; approaches to validate or make adjustments (such as half cycle corrections) to a model; and methods for handling population heterogeneity and uncertainty.		
Results				
Study parameters	18	Report the values, ranges, references, and, if used, probability distributions for all parameters. Report reasons or sources for distributions used to represent uncertainty where appropriate. Providing a table to show the input values is strongly recommended.		
Incremental costs and outcomes	19	For each intervention, report mean values for the main categories of estimated costs and outcomes of interest, as well as mean differences between the comparator groups. If applicable, report incremental cost effectiveness ratios.		
Characterising uncertainty	20a	Single study-based economic evaluation: Describe the effects of sampling uncertainty for the estimated incremental cost and incremental effectiveness parameters, together with the impact of methodological assumptions (such as discount rate, study perspective).		
	20b	Model-based economic evaluation: Describe the effects on the results of uncertainty for all input parameters, and uncertainty related to the structure of the model and assumptions.		
Characterising heterogeneity	21	If applicable, report differences in costs, outcomes, or cost effectiveness that can be explained by variations between subgroups of patients with different baseline characteristics or other observed variability in effects that are not reducible by more information.		
Discussion				
Study findings, limitations, generalisability, and current knowledge	22	Summarise key study findings and describe how they support the conclusions reached. Discuss limitations and the generalisability of the findings and how the findings fit with current knowledge.		
Other				
Source of funding	23	Describe how the study was funded and the role of the funder in the identification, design, conduct, and reporting of the analysis. Describe other non-monetary sources of support.		
Conflicts of interest	24	Describe any potential for conflict of interest of study contributors in accordance with journal policy. In the absence of a journal policy, we recommend authors comply with International Committee of Medical Journal Editors recommendations

**Table 4:** Philips criterion [25, 26]

Dimensions of quality	Questions for critical appraisal	Response (Yes/no/partial/unclear/NA)	Comments
S1: Statement of decision problem/objective	Is there a clear statement of the decision problem?		
	Is the objective of the evaluation and model specified and consistent with the stated decision problem?		
	Is the primary decision maker specified?		
S2: Statement of scope/perspective	Is the perspective of the model stated clearly?		
	Are the model inputs consistent with the stated perspective?		
	Has the scope of the model been stated and justified?		
	Are the outcomes of the model consistent with the perspective, scope and overall objective of the model?		
S3: Rationale for structure	Has the evidence regarding the model structure been described?		
	Is the structure of the model consistent with a coherent theory of the health condition under evaluation?		
	Have any competing theories regarding model structure been considered?		
	Are the sources of data used to develop the structure of the model specified?		
	Are the causal relationships described by the model structure justified appropriately?		
S4: Structural assumptions	Are the structural assumptions transparent and justified?		
	Are the structural assumptions reasonable given the overall objective, perspective and scope of the model?		
S5: Strategies/comparators	Is there a clear definition of the options under evaluation?		
	Have all feasible and practical options been evaluated?		
	Is there justification for the exclusion of feasible options?		
S6: Model type	Is the chosen model type appropriate given the decision problem and specified causal relationships within the model?		
S7: Time horizon	Is the time horizon of the model sufficient to reflect all important differences between options?		
	Is the time horizon of the model, and the duration of treatment and treatment effect described and justified?		
	Has a lifetime horizon been used? If not, has a shorter time horizon been justified?		
S8: Disease states/pathways	Do the disease states (state transition model) or the pathways (decision tree model) reflect the underlying biological process of the disease in question and the impact of interventions?		
S9: Cycle length	Is the cycle length defined and justified in terms of the natural history of disease?		
D1: Data identification	Are the data identification methods transparent and appropriate given the objectives of the model?		
	Where choices have been made between data sources, are these justified appropriately?		
	Has particular attention been paid to identifying data for the important parameters in the model?		
	Has the process of selecting key parameters been justified and systematic methods used to identify the most appropriate data?		
	Has the quality of the data been assessed appropriately?		
	Where expert opinion has been used, are the methods described and justified?		
D2: Pre-model data	Are the pre-model data analysis methodology based on justifiable statistical and epidemiological techniques?		
D2a: Baseline data	Is the choice of baseline data described and justified?		
	Are transition probabilities calculated appropriately?		
	Has a half cycle correction been applied to both cost and outcome?		
D2b: Treatment effects	If relative treatment effects have been derived from trial data, have they been synthesised using appropriate techniques?		
	Have the methods and assumptions used to extrapolate short-term results to final outcomes been documented and justified? Have alternative assumptions been explored through sensitivity analysis?		
	Have assumptions regarding the continuing effect of treatment once treatment is complete been documented and justified? Have alternative assumptions been explored through sensitivity analysis?		
D2c: Quality-of-life weights (utilities)	Are the utilities incorporated into the model appropriate?		
	Is the source for the utility weights referenced?		
	Are the methods of derivation for the utility weights justified?		
D3: Data incorporation	Have all data incorporated into the model been described and referenced in sufficient detail?		
	Has the use of mutually inconsistent data been justified (i.e. are assumptions and choices appropriate)?		
	Is the process of data incorporation transparent?		
	If data have been incorporated as distributions, has the choice of distribution for each parameter been described and justified?		
D4: Assessment of uncertainty	Have the four principal types of uncertainty been addressed?		
	If not, has the omission of particular forms of uncertainty been justified?		
D4a: Methodological	Have methodological uncertainties been addressed by running alternative versions of the model with different methodological assumptions?		
D4b: Structural	Is there evidence that structural uncertainties have been addressed via sensitivity analysis?		
D4c: Heterogeneity	Has heterogeneity been dealt with by running the model separately for different sub-groups?		
D4d: Parameter	Are the methods of assessment of parameter uncertainty appropriate?		
	Has probabilistic sensitivity analysis been done, if not has this been justified?		
	If data are incorporated as point estimates, are the ranges used for sensitivity analysis stated and justified?		
C1: Internal consistency	Is there evidence that the mathematical logic of the model has been tested thoroughly before use?		
C2: External consistency	Are the conclusions valid given the data presented?		
	Are any counterintuitive results from the model explained and justified?		
	If the model has been calibrated against independent data, have any differences been explained and justified?		
	Have the results of the model been compared with those of previous models and any differences in results explained?		

To initially assess the quality of included studies, a score of 1 will be given to items within the CHEERS checklist that can be answered ‘yes’, 0.5 for items only partially addressed, 0 for ‘no’ and where an item is identified as not applicable, no score will be given. The score will then be totalled and calculated as a percentage of the total score that could be achieved for that study, thus taking into account elements of the checklist which were deemed not applicable. This is an approach that has previously been used [27], and although it is acknowledged that by assigning equal weighting to each criteria may not truly reflect the importance of each of the checklist items, it is thought that it will provide a broad overview of the quality of studies included, which will then pave way for more detailed analysis on individual checklist items. These evaluations will be included in any publication as supplementary material where feasible.

Methodological variation between studies is likely to prevent a pooling of economic data in the form of a meta-analysis, and therefore, results of the studies will be presented and discussed in a qualitative manner according to the study type.

## Discussion

This systematic review will provide a comprehensive assessment of the type and quality of economic research used in the evaluation of interventions to prevent and treat eczema. The results of the review are likely to be written up in multiple publications, one focusing on an overview of the state of the art with additional papers focusing in more detail on particular methodologically aspects (for instance, the methods used in modelling studies). The review will report the range of cost-effectiveness estimates found for interventions to prevent and treat atopic eczema, which may be useful in informing clinicians and decision makers about the relative value of different interventions for eczema and enable the value of eczema interventions to be compared with the cost effectiveness for other interventions in other disease areas. That is, it may help decision makers, on the basis of current information (if sufficient), to be able to answer questions about how to allocate limited resources between eczema and other disease areas and once allocated to eczema how to use those limited resources efficiently to maximise outcomes from eczema care. The review will also be of interest to methodologists interested in the range and quality of economic studies in this clinical field. Finally, this systematic review will help identify gaps in the current evidence base surrounding the economics of eczema to inform further research efforts in this area.

## Appendix

### Details of electronic bibliographic database search strings

Medline (1946 to present) & Embase (1974 to present) (via Ovid, http://ovidsp.tx.ovid.com/)

1. (dermatitis or eczema).tw.
2. exp Eczema/
3. exp Dermatitis, Atopic/
4. exp Economics/
5. exp Quality‐Adjusted Life Years/
6. exp Health Care Costs/
7. (econ* or cost* or price* or expenditure* or "pharmacoecon*" or budget* or "value of life" or qaly* or "quality adj* life year*" or utilit* or pricing* or "net benefit*" or "monetary benefit*" or "net health benefit*" or "willingness to pay" or "fee*” or “charge*").tw.
8. 1 or 2 or 3
9. 4 or 5 or 6 or 7
10. 8 and 9

Web of Science (various indexes included earliest start date 1970 to present) (http://apps.webofknowledge.com)

1. TS=(dermatitis or eczema)
2. TS=((econ* or cost* or price* or expenditure* or "pharmacoecon*" or budget* or "value of life" or qaly* or "quality adj* life year*" or utilit* or pricing* or "net benefit*" or "monetary benefit*" or "net health benefit*" or "willingness to pay" or "fee*” or “charge*"))
3. 1 and 2

EconLit (1886 to present) and CINAHL (1937 to present) (via EBSCO, http://web.a.ebscohost.com)

1. MH "Dermatitis, Atopic" OR MH "Eczema"
2. AB Dermatitis OR Eczema
3. (MH "Economics+") OR (MH "Health Resource Allocation") OR (MH "Health Care Costs+") OR (MH "Health Care Delivery+") OR (MH "Quality Adjusted Life Years") OR (MH "Quality of Life") OR (MH "Economic Value of Life")
4. AB econ* or cost* or price* or expenditure* or "pharmacoecon*" or budget* or "value of life" or qaly* or "quality adj* life year*" or utilit* or pricing* or "net benefit*" or "monetary benefit*" or "net health benefit*" or "willingness to pay" or “fee*” or “charge*”
5. 1 or 2
6. 3 or 4
7. 5 and 6

Scopus (1960 to present) (http://www.scopus.com)

1. (TITLEABSKEY (dermatitis OR eczema)) AND (TITLEABSKEY ((econ* OR cost* OR price* OR pricing* OR expenditure* OR "pharmacoecon*" OR budget* OR "value of life" OR qaly* OR "quality adj* life year*" OR utilit* OR "net benefit*" OR "monetary benefit*" OR "net health benefit*" OR "willingness to pay" OR "fee*” OR “charge*")))

Cochrane Database of Systematic Reviews/Database of Abstracts of Reviews of Effect/Cochrane Central Register of Controlled Trials/Health Technology Assessment Database/NHS Economic Evaluation Database (all years searched)

(via Wiley, http://onlinelibrary.wiley.com/cochranelibrary/search/)

1. dermatitis or eczema.ti.ab
2. MeSH descriptor: [Dermatitis, Atopic] explode all trees OR MeSH descriptor: [Eczema] explode all trees
3. econ* or cost* or price* or expenditure* or "pharmacoecon*" or budget* or "value of life" or qaly* or "quality adj* life year*" or utilit* or pricing* or "net benefit*" or "monetary benefit*" or "net health benefit*" or "willingness to pay" or "fee*” OR “charge*".ti.ab.
4. MeSH descriptor: [Economics] explode all trees OR MeSH descriptor: [Quality Adjusted Life Years] explode all trees OR MeSH descriptor: [Health Care Costs] explode all trees
5. 1 or 2
6. 3 or 4
7. 5 and 6

CEA Registry (1976 to present) (https://research.tufts-nemc.org/cear4/Home.aspx)

1. Eczema
2. Dermatitis

## Additional file

Additional file 1: Preferred Reporting Items for Systematic review and Meta-Analysis Protocols (PRISMA-P) 2015 checklist: recommended items to address in a systematic review protocol. (DOCX 15kb)

## Abbreviations

CHEERS: Consolidated Health Economic Evaluation Reporting Standards
GP: General Practitioner
GREAT: Global Resource for Eczema Trials
HQoL: Health-Related Quality of Life
ICER: Incremental Cost Effectiveness Ratio
JLA: James Lind Alliance
NA: Not Applicable
NHS: National Health Service
NICE: National Institute of Health and Care Excellence
PSP: Priority Setting Partnership
QALYs: Quality Adjusted Life Years
UK: United Kingdom
